# Performance Study of Waste PE-Modified High-Grade Asphalt

**DOI:** 10.3390/polym15153200

**Published:** 2023-07-27

**Authors:** Erda Li, Wenyuan Xu, Yang Zhang

**Affiliations:** 1 College of Civil Engineering, Northeast Forestry University, Harbin 150040, China; 2 Heilongjiang Provincial Transportation Investment Group, Harbin 150036, China

**Keywords:** waste PE, modified asphalt, rheological properties, thermal properties, modification mechanism

## Abstract

In this work, waste polyethylene (PE)-modified 90# asphalt was made in order to investigate the performance of waste polyethylene-modified high-grade asphalt and the optimal blending quantity. Dynamic Shear Rheology (DSR) and Bending Beam Rheometer (BBR) tests were used to evaluate the high- and low-temperature performance of modified 90# PE-modified asphalt. Infrared spectroscopy and fluorescence microscopy were used to investigate the modification process and distribution status of waste PE in 90# asphalt. The DSR and BBR tests revealed that waste PE enhanced the high-temperature performance of 90# base asphalt and that 5% was the best blending rate. However, the change affects asphalt’s low-temperature performance, and the negative effect on asphalt’s low-temperature performance was minimized at 1% dosing. The incorporation of waste PE absorbed the light components of asphalt, while waste PE can form a reticulated structure in asphalt, which improves its high-temperature performance but degrades its low-temperature performance, according to the results of infrared spectroscopy and fluorescence microscopy.

## 1. Introduction

In today’s world, sustainable development is gradually becoming a common goal pursued by all countries. In daily life, people consume a lot of plastic products. According to statistics, China’s current annual plastic waste volume reaches 38.4 million tons, accounting for 42.3% of plastic usage. The haphazard accumulation of discarded plastic is unavoidably harmful to the ecosystem. As a result, recycling waste plastics will effectively solve this issue.

For the recycling of waste plastics, researchers have made many efforts and applied them to several industries [1,2,3]. Passamonti F. J. et al. applied used low-density polyethylene (LDPE) to gas oil to investigate the effect of the application of used LDPE on the yield of different products from gas oil. The study’s conclusions indicate that adding waste LDPE can significantly enhance the generation of dry gas and gasoline and enable the use of waste PE in fuel [4]. Soni V. K. et al. evaluated the research on waste PE in the field of pyrolysis and analyzed the use of pyrolysis oil as a fuel or fuel additive [5]. Wei et al. applied waste PE to lithium battery applications and prepared new high-value-added carbon nanomaterials for LIBs [6]. In terms of organic reuse from waste plastics, Wang C Q et al. extracted ethylene glycol benzodicarboxylate from urban waste plastics and achieved a high purification of 98.46%, enabling the use of waste plastics in industry [7].

Waste PE has also been widely used in civil engineering [8,9,10]. This is reflected in its application in concrete [11], retaining walls [12], water engineering [13], and other applications. By partially replacing the sand in concrete with waste plastics, Ismail Z. Z. et al. investigated the effects of different replacement rates on the properties of concrete. The study’s conclusions showed that a partial replacement of sand with waste plastic might effectively stop the growth of microcracks in concrete while also saving money [14]. Al-Hadithi A. I. et al. created self-compacting concrete using waste plastic fibers and researched the impact of waste plastics on the concrete’s ability to harden. The results of the investigation revealed that adding waste plastics can greatly increase concrete’s compressive and flexural strength [15]. Khan M et al. studied the impact of recycled plastic pins (RPP) on preventing retaining wall sliding by employing recycled plastics placed in temporary retaining wall foundations. The study’s findings revealed that recycled plastic pegs can provide anti-slip resistance and prevent displacement at the retaining wall’s base [16]. Galvo J. C. A. et al. used waste plastics to repair dam materials. The study’s findings revealed that the repair materials generated from a 2.5% mixing of waste plastics had the best strength and underwater erosion and abrasion resistance qualities [17].

Researchers are also gradually applying waste plastics to asphalt pavements in order to achieve significant recycling of waste plastics. Haider S. et al. prepared asphalt mixtures with high-density polyethylene and assessed moisture damage performance. The study’s findings revealed that adding waste plastics to asphalt mixes can successfully prevent moisture damage to asphalt mixes [18]. Meanwhile, White G’s research discovered that asphalt coated with waste plastic can considerably increase the asphalt’s resistance to deformation [19]. Changqing et al. investigated how waste polyethylene packaging treated with asphalt performed. According to the findings of the investigation, the best service life of the modified asphalt was obtained when the waste polyethylene packaging was combined at less than 10% [20]. Boom Y J et al. conducted research on the performance of recycled-plastic-modified asphalt mixes. The study’s findings revealed that modifying asphalt mixes with recycled plastics increased their fatigue and rutting resistance. It also helps lower gas emissions during the manufacture of asphalt mixtures [21]. Ren S et al. opted to add Reactive Ethylene Terpolymer (RET) to PE-modified asphalt to improve the elastic characteristics of the modified asphalt in order to improve the overall performance of the modified asphalt, and the optimal RET doping was found to be 1 wt% [22].

Scholars have also conducted studies on modified asphalt after aging. Ma Y et al., for example, investigated the aging performance and anti-aging mechanism of a rubber-polyethylene composite-modified asphalt. The aging of rubber–PE composite-modified asphalt in the environment was discovered to be more severe than that under pressure. This aging was caused by the PE [23]. Ren S et al. also developed a kinetic model for the long-term aging response of asphalt in order to anticipate the internal chemical changes in asphalt over time [24]. Furthermore, Xiao R et al. investigated the performance of asphalt mixes following the PE treatment of the aggregate surface. The study’s findings revealed that treating the surface of acidic aggregates with LDPE may significantly increase the adhesion qualities of asphalt and acidic aggregates. The best adhesion qualities between asphalt and aggregate follow HDPE surface treatment [25].

The performance of PE-modified asphalt and its mixtures is also affected by different preparation techniques. Currently, two processes for producing PE-modified asphalt are available: dry and wet. Existing study results demonstrate that when PE-modified asphalt is chosen for wet preparation, the modifier rapidly separates from the asphalt, resulting in poor storage stability of PE-modified asphalt after wet preparation [26]. The majority of studies have used the dry preparation of PE-modified asphalt and asphalt mixtures. Pasetto M et al., for example, created asphalt mixtures with various PE blends using the dry process and examined their performance. The study’s findings revealed that the dry preparation of molded asphalt mixes improves the mechanical characteristics and moisture resistance [27]. As a result, the dry approach was also adopted in this study for the manufacture of waste PE-modified asphalt.

Therefore, according to previous research, waste PE can be applied to asphalt pavement. The study on enhancing high-grade asphalt with PE is still restricted, and the majority of studies on PE-modified asphalt are focused on increasing 70# base asphalt’s high-temperature performance. Existing research focuses on the modification of 70# asphalt by waste PE since 70# asphalt has higher high-temperature performance and is mostly utilized in hot climates. The addition of waste PE to 70# asphalt improves its high-temperature performance. performance and is mostly employed in high-temperature environments. However, its low-temperature performance is inferior to that of 90# matrix asphalt, which is mostly employed in low-temperature locations. Although high-grade asphalt is more commonly utilized in low-temperature areas, as the environment warms and pavements are subjected to increasingly heavy loads, both call for low-temperature pavements to have higher heat-induced deformation resistance. Based on the above-mentioned problems, this study opted to alter the 90# base asphalt widely used in low-temperature locations by blending it with waste PE, resulting in high-grade modified asphalt that meets both low-temperature and enhanced high-temperature performance. DSR and BBR studies were utilized to assess the changed asphalt’s high- and low-temperature performance and the modification process and mechanism were discovered by combining infrared spectroscopy and fluorescence microscopy investigations. The study was carried out to investigate the practicality of employing waste PE in road building in cold areas.

## 2. Material Preparation and Test Methods

### 2.1. Raw Materials

The 90# matrix asphalt utilized in this research was sourced from Jining, Shandong Province. Table 1 shows its technical indications after testing:

The data in Table 1 show that, after testing, the properties of the selected 90# base asphalt meet the performance requirements.

Testing revealed that the waste PE used in this paper is low-density polyethylene (LDPE), the key characteristics of which are listed in Table 2.

### 2.2. Method of Preparing PE-Modified Asphalt

Making PE uniformly diffused in asphalt is the key to the preparation method for PE-modified asphalt. Related studies have shown that PE is best dispersed in asphalt when the temperature is 50 °C above the PE melting point [28]. Simultaneously, in order to compare the modification effects of various PE dopings, this paper mixed 1%, 2%, 3%, 4%, 5%, and 6% of the weight of asphalt with PE modifier. As a result, the asphalt mixed with polyethylene in this paper was sheared at a high speed for 1 h at 175 °C to ensure that the polyethylene was uniformly dispersed in the asphalt. It was then baked for 20 min at 175 °C to decrease the impact of internal air bubbles on the test results.

### 2.3. Attenuated Total Reflection Infrared Spectroscopy Tests (ATR-FTIR)

In this paper, in order to test the PE-modified asphalt functional group without damaging the specimen, the test was conducted by the attenuated total reflection method. The test samples were PE-modified asphalt with 0%, 1%, 2%, 3%, 4%, 5%, and 6% admixtures. The weights of the test samples were all 0.5 g. The tests in this work were carried out using a Thermo Fisher Scientific Nicolet-iS5 FTIR spectrometer (Waltham, MA, USA) at infrared wavelengths ranging from 600 cm^−1^ to 4000 cm^−1^.

### 2.4. Dynamic Shear Rheology Tests (DSR)

**Figure 1:**
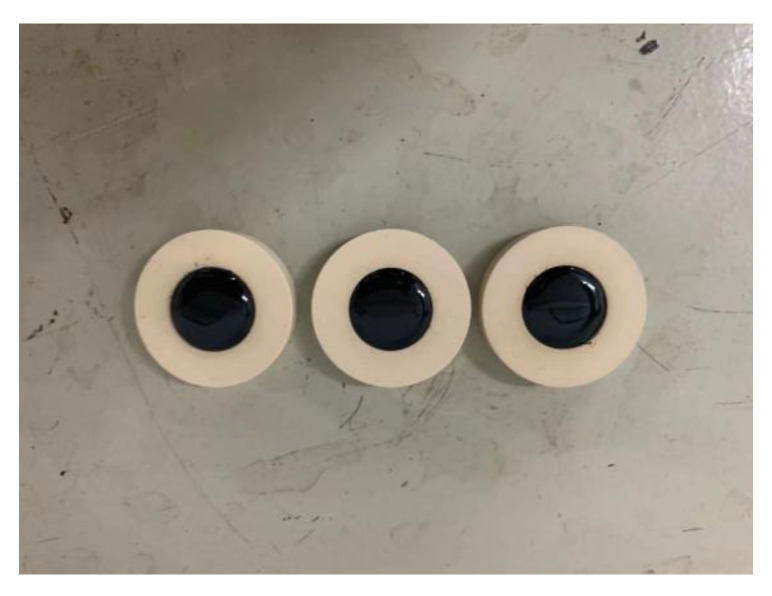
Asphalt DSR specimens to be tested.

The DSR test can evaluate asphalt’s high-temperature viscoelastic characteristics [29]. In this investigation, DSR tests were used to measure the dynamic shear modulus (|G∗||) and phase angle (δ) of asphalt treated with PE. In order to compare the test results with 90# base asphalt and SBS-modified asphalt, the test temperatures were 46 °C, 52 °C, 58 °C, 64 °C, 70 °C, 76 °C, 82 °C, and 88 °C. Three samples of each asphalt were tested at each temperature in order to reduce the impact of test errors, and the results were averaged. The prepared DSR specimens are shown in Figure 1.

### 2.5. Bending Beam Rheometer Tests (BBR)

The BBR test can evaluate asphalt’s low-temperature capabilities [30]. In this research, the low-temperature performance of PE-modified asphalt was examined at −18 °C and −24 °C. The applied load was 980 ± 5 mN, and three specimens of each doping were tested at the same temperature to avoid the effect of test errors. Equations (1) and (2) were used to compute the bending creep stiffness S and creep rate m.

(1) S(t)=PL34bh3δ(t)

(2) $$m(t)=B+2C\left|lg\left(t\right)\right|$$

where S(t) is the creep stiffness, MPa; m(t) is the creep rate; P is the applied load during the measurement, mN; L is the span length, mm; b is the depth of the specimen, mm; h is the depth of the specimen, mm; δ(t) is the deflection of the specimen; and A, B, C is the regression coefficient.

**Figure 2:**
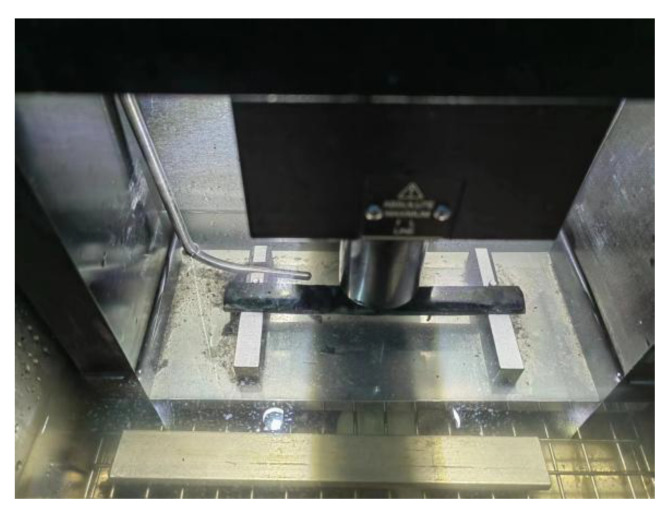
Test procedure of asphalt BBR test.

Figure 2 depicts the BBR test procedure.

### 2.6. Fluorescence Microscope Tests (FM)

This research chose to test utilizing FM with a magnification of 10 times during the test to assess the distribution of modifiers in asphalt at different doses.

## 3. Results and Discussions

### 3.1. Analysis of ATR-FTIR Results

**Figure 3:**
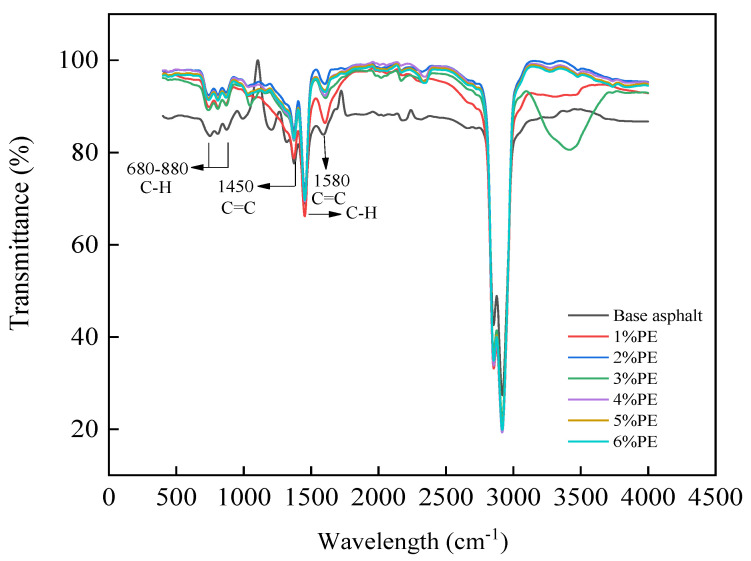
Infrared spectroscopy results of modified asphalt with different PE doses.

In this paper, ATR-FTIR was used to reveal the effect on the chemical composition of asphalt at different PE doping levels, and the IR spectra obtained after the tests are shown in Figure 3.

**Figure 4:**
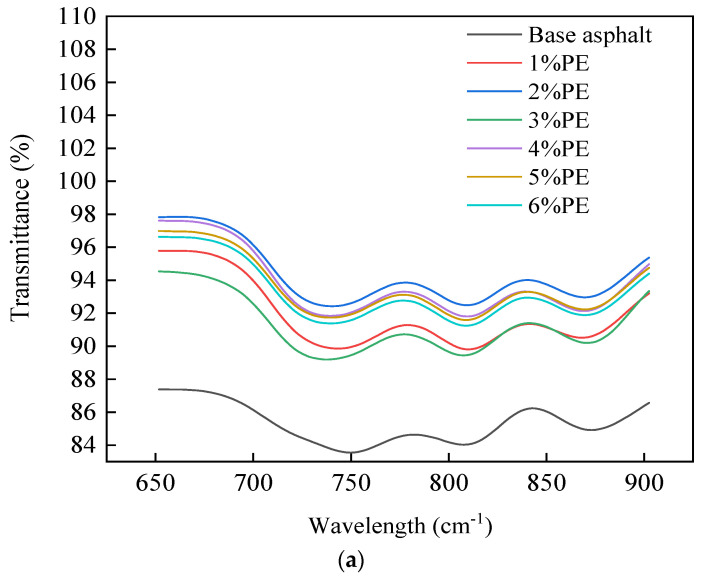

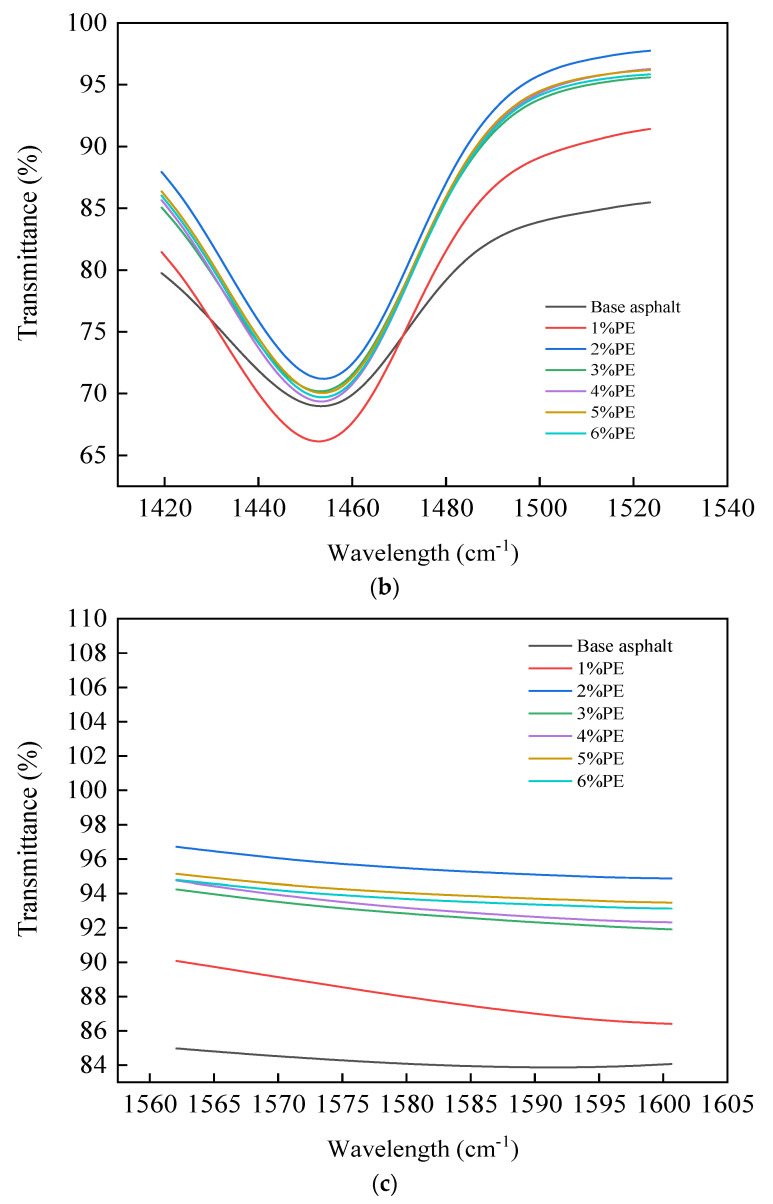
Infrared spectra after intercepting different wavelength ranges. (**a**) Infrared spectra at 650 cm^−1^–900 cm^−1^. (**b**) Infrared spectrum in the range of 1420 cm^−1^–1520 cm^−1^. (**c**) IR spectrum in the range 1560 cm^−1^–1595 cm^−1^.

As can be seen in Figure 3, the transmittance of the different peaks at 680 cm^−1^–880 cm^−1^, 1450 cm^−1^, 1500 cm^−1^, and 1580 cm^−1^ varied significantly. Therefore, the local infrared spectra at the above peak were selected as shown in Figure 4:

Figure 4 illustrates that as PE doping increases, the transmittance undergoes a dynamic change process. The change in the concentration of characteristic functional groups can be reflected in the transmittance, and the greater the transmittance, the lower the concentration of functional groups. Meanwhile, the characteristic peaks at 680–880 cm^−1^, 1450 cm^−1^, 1500 cm^−1^, and 1580 cm^−1^ in aromatic hydrocarbons correspond to the C-H out-of-plane distortion vibration, C=C skeletal vibration, C-H vibration, and C=C vibration, respectively. As a result, the addition of PE induces a change in the aromatic fraction concentration of the asphalt fraction. However, the permeability reached its maximum at 2%, decreased at 3%, and then gradually increased with the increase in dosing. This paper speculates that the reason for this phenomenon is related to the distribution state of waste PE in asphalt. At 2% admixture, a more obvious chain structure was not formed in the asphalt, and the waste PE had more surface area in the asphalt to absorb the lighter components.

The concentration of characteristic functional groups of aromatic fractions is higher when no modifier is added. With the addition of PE, the concentration of characteristic functional groups in the aromatic fraction showed a tendency to decrease. This is related to the absorption of light components in asphalt by blending in PE, such as the aromatic content.

The distinctive peaks of the infrared spectra can represent the magnitude of the functional group concentration [31]. There are peak variances at 680–880 cm^−1^ and 1450 cm^−1^ for different waste PE doses, as shown in Figure 4a,b. When 2% waste PE was added, the transmission rate of C-H out-of-plane distortion vibration increased by 9% compared to unmatrixed bitumen. When 5% waste PE was mixed in, the transmittance of C-H out-of-plane distortion vibration increased by 7.8%. When 2% PE was added to base asphalt, the transmission rate of C=C skeletal vibration increased by 4%. At 5% waste PE doping, the transmittance of C=C skeletal vibration increased by 3.2%. A reduction in the concentration of the relevant functional group is indicated by an increase in permeability. This confirmed the hypothesis that blending waste PE absorbs the lighter components of asphalt.

### 3.2. Analysis of High-Temperature Performance Test Results

The dynamic shear modulus (|G^∗^|) and phase angle (δ) of asphalt can show its high-temperature performance. Temperature scans of modified asphalt, 90# matrix asphalt, and SBS-modified asphalt with various PE admixtures were performed utilizing DSR experiments in this paper. During the test, the strain control mode was used. The test temperatures were 46 °C, 52 °C, 58 °C, 64 °C, 70 °C, 76 °C, 82 °C, and 88 °C to analyze the effect of PE on the high-temperature performance of asphalt. The control strain during the test was 12%.

**Figure 5:**
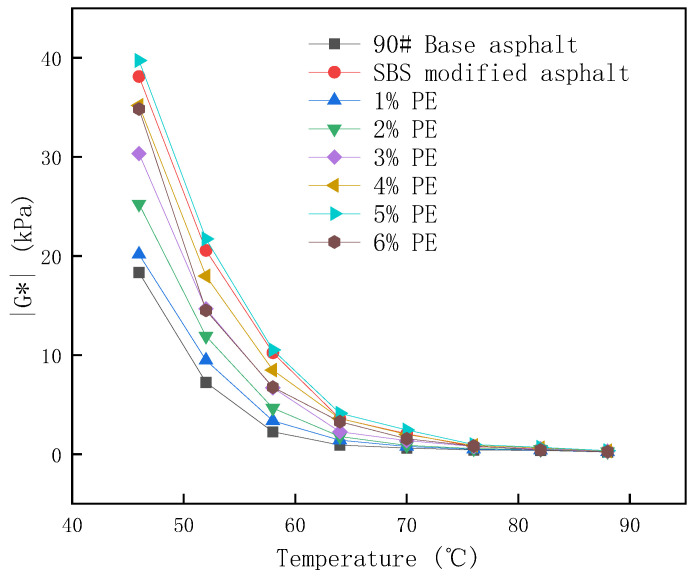
Test results of complex shear modulus.

Figure 5 depicts the results of the complicated shear modulus test.

According to the findings in Figure 5, matrix asphalt has the lowest complicated shear modulus. The complex shear modulus steadily increases with increasing PE dose, reaching a maximum at 5% dosing and decreasing at 6% dosing. The complex shear modulus was 116% increased over base asphalt at 46 °C. The complex shear modulus can reflect the capability of asphalt to resist distortion, implying that the addition of waste PE can effectively improve the matrix asphalt’s high-temperature deformation resistance. This is due to the fact that PE in asphalt can absorb the lighter components of the asphalt and produce swelling that binds the movement of the asphalt. The complex shear modulus decreases at a doping level of 6%. This paper speculates that this is due to the fact that the lighter components in the asphalt cannot effectively fuse with the polyethylene in the waste PE due to the excessive amount of PE blending, which results in a smaller complex shear modulus at 6% blending than that at 5% blending. Meanwhile, the data in Figure 5 demonstrate that at temperatures less than 60 degrees Celsius, the high-temperature deformation resistance of 5% PE-modified asphalt is superior to that of SBS-modified asphalt.

**Figure 6:**
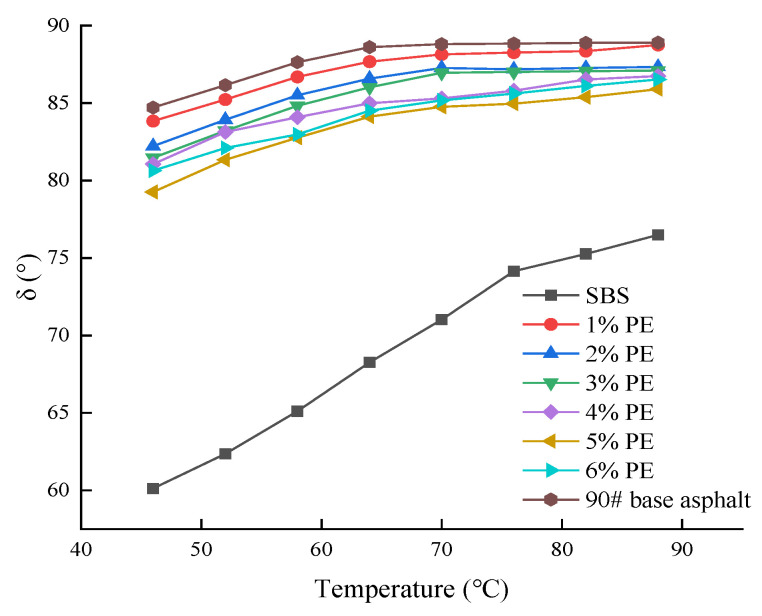
Phase angle test results for different asphalts.

Figure 6 depicts the phase angle test findings.

**Figure 7:**
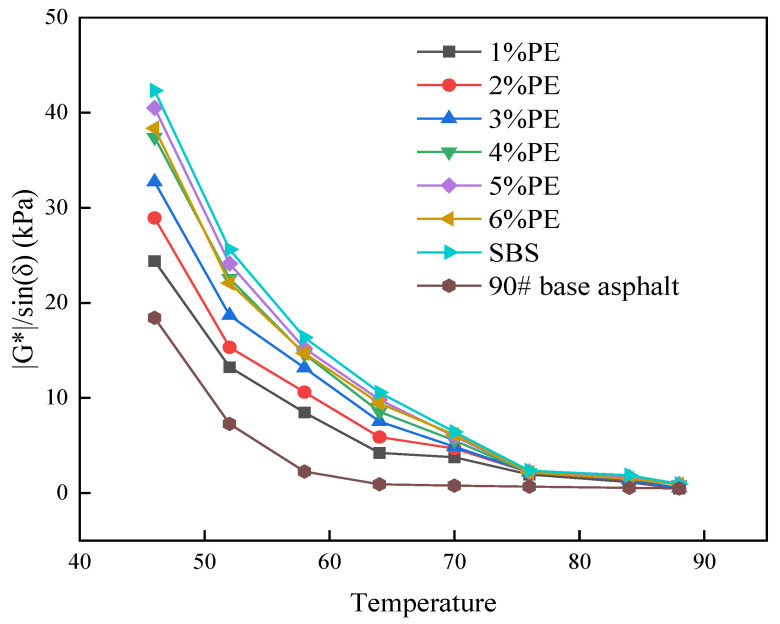
Test results of different asphalt rutting factors.

The data in Figure 6 show that the phase angles of different types of asphalt show an increasing trend as the temperature increases. The phase angle dropped significantly as the PE doping level increased from 1% to 5%. For both PE-modified asphalt and SBS-modified asphalt, the phase angle increased by 5% to 6% admixture and was smaller than 90# matrix-modified asphalt. The phase angle might reflect the viscoelasticity of the asphalt; the bigger the phase angle, the less elastic and deformable the asphalt. As a result, it can be concluded that the addition of waste PE can increase the elastic properties of asphalt. Among them, the dose of 5% to improve the elastic properties of asphalt was the best, but the elastic qualities of PE-modified asphalt were still inferior to SBS-modified asphalt at the time. Figure 7 depicts the results of the rutting factor tests.

The rutting factor can indicate asphalt’s high-temperature performance; the higher the rutting factor, the better the asphalt’s high-temperature performance. According to the results in Figure 7, the rutting factors of various types of modified asphalt are bigger than those of 90# matrix asphalt. This implies that the inclusion of the modification improves the asphalt’s high-temperature performance. At the same temperature, the rutting factor of PE-modified asphalt in the 1% to 5% admixture range increased, whereas the rutting factor of PE-modified asphalt at 6% admixture dropped. This shows that at this doping level, the high-temperature performance of PE-modified asphalt was reduced, which is consistent with the trend illustrated in Figure 5 and Figure 6. Simultaneously, the rutting factor calculation results showed that the 5% dose of PE-modified asphalt with high-temperature performance was the best and was very near to the performance of SBS-modified asphalt. As a result, it can be concluded that the high-temperature performance of waste plastics used to alter asphalt at 5% admixture can be comparable to that of SBS-modified asphalt. It may be utilized as an alternative to SBS modifiers and can successfully minimize waste plastic pollution.

Also in this paper, the temperature sensitivity of PE-modified asphalt at different dosages was calculated based on Equation (3):

LgG∗ = K × T + b(3)

**Figure 8:**
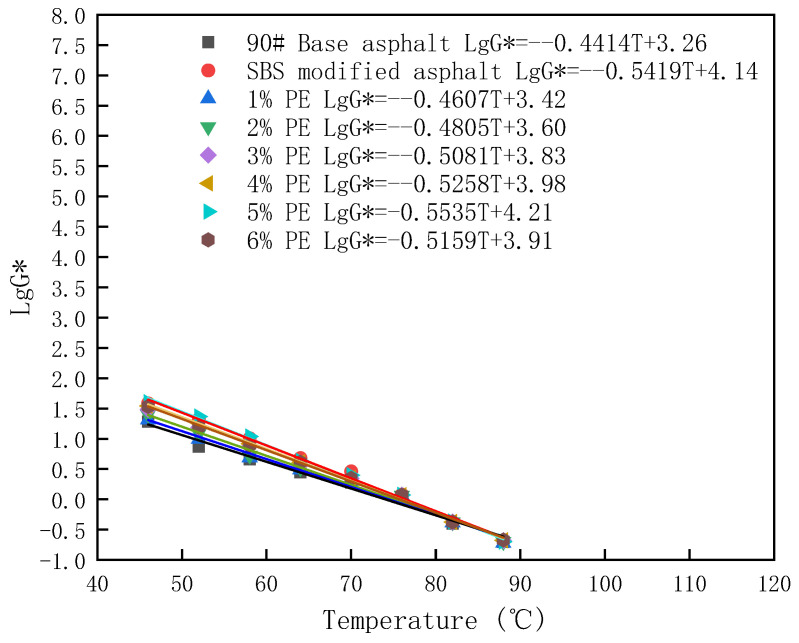
Temperature sensitivity of asphalt at different types and dosages.

Related studies have shown that the sensitivity to temperature increases when the absolute value of K is larger [32]. The results of the calculations in this paper are shown in Figure 8.

From the calculations in Figure 8, it can be found that when the doping is 5%, the absolute value of K is maximum. Therefore 5% PE-modified asphalt has the highest temperature sensitivity. This is related to the network structure formed by PE in asphalt at 5% PE dosage. The formation of the mesh structure is not favorable to the temperature sensitivity of asphalt. This is consistent with the findings of Wen Y et al. [33].

### 3.3. Analysis of Low-Temperature Performance Test Results

In this study, the BBR test was used to assess the influence of PE admixture on the low-temperature performance of asphalt using two indexes: modulus of stiffness (S) and creep rate (m).

**Figure 9:**
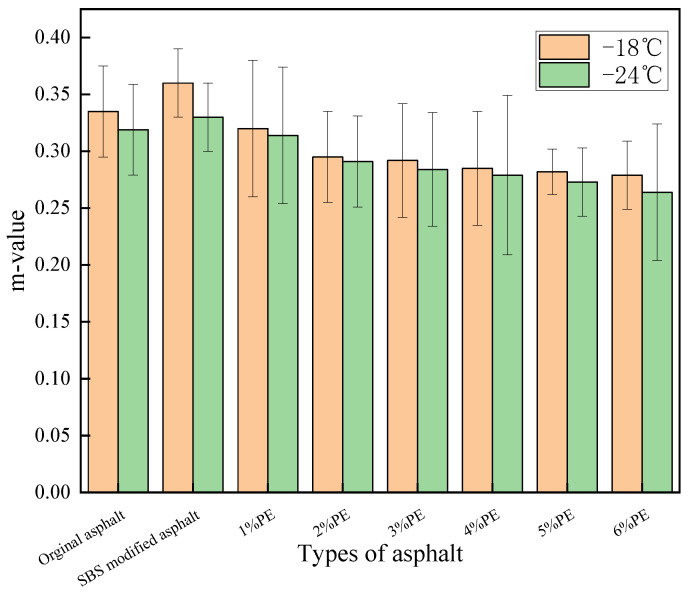
Creep rate test results of different asphalts.

Figure 9 depicts the creep rate (m) test results.

The greater the creep rate (m), the slower the building of thermal stresses in the modified asphalt and the less influence on pavement cracking. The measurements in Figure 9 show that the creep rate of asphalt steadily decreases as the amount of PE additive increases. The m-values corresponding to −18 °C and −24 °C were 0.279 and 0.264, respectively, at 6% admixture, while the m-values of the matrix asphalt at these two temperatures were 0.335 and 0.319, respectively. Compared with the matrix asphalt, the m-value of 6% PE-modified asphalt decreased by 16.7% and 17.2%, respectively. The decrease in creep rate is likewise related to the dissolution of PE in asphalt. After PE was mixed into asphalt, there was physical co-mingling and swelling in the asphalt as well as certain chemical reactions, making the dispersed PE cross-linked to form a chain-like structure. And, when the PE admixture grew, the PE chain structures were cross-linked with each other to form a mesh structure, which inhibited the creep of asphalt under temperature stress and other loads, readily resulting in brittle damage of the asphalt, lowering the crack resistance of the asphalt.

Meanwhile, the value of m was close to the base asphalt when PE doping was 1%, and the value of m was greater than 0.3 at both −18 °C and −24 °C at this time. As a result, if we want to increase both high- and low-temperature performance, we can pick waste PE with 1% doping.

When the m-values of SBS and waste PE-modified asphalt were compared, the creep rate of waste PE-modified asphalt was found to be smaller than that of SBS-modified asphalt. This is related to the difference in the role of SBS and PE in asphalt. SBS in asphalt has just a physical co-blending action, and its enhancement of low-temperature qualities is generated by the movement of the hard segments attaching the asphalt at both ends of the SBS molecule. When PE is mixed with asphalt, there is both physical co-mingling and a chemical reaction of PE molecules cross-linking with each other. At a dosing rate of more than 2%, a chain structure was created in the asphalt and gradually cross-linked into a mesh structure, thus producing a better binding effect on the asphalt compared to the SBS modification. Therefore, it is more prone to brittle fracture than SBS-modified asphalt.

When the values of m of PE-modified asphalt at −18 °C and −24 °C with different blends are compared, it is discovered that the drop in temperature has a negative effect on the fracture resistance of the PE-modified asphalt. This is related to the nature of the asphalt. A drop in temperature will cause the asphalt to become hard as well as brittle, thus reducing the cracking resistance of the modified asphalt.

**Figure 10:**
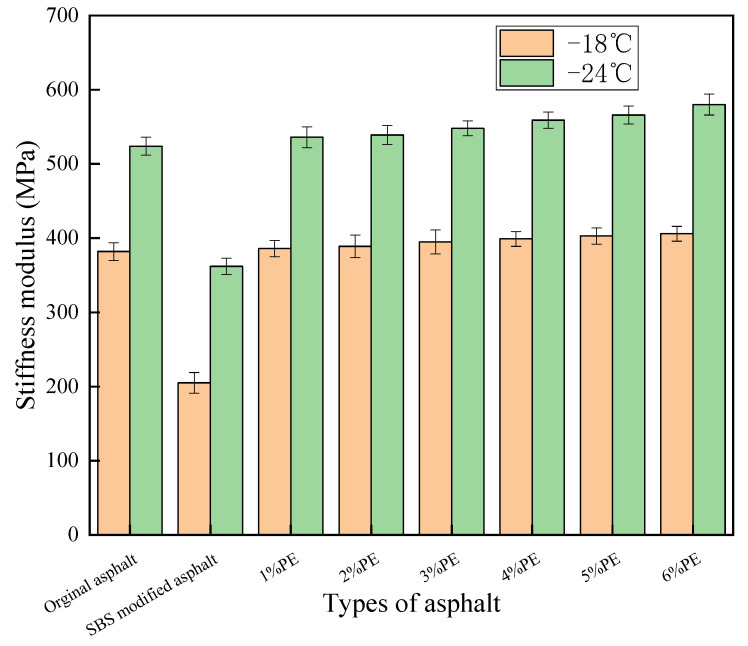
Modulus of stiffness of different types of asphalt.

The results of creep stiffness (S) tests are shown in Figure 10:

The modulus of stiffness reflects the asphalt’s capacity to withstand loads. The higher the modulus of stiffness, the lower the asphalt’s low-temperature fracture resistance. Figure 10 shows that the addition of PE enhances the stiffness modulus of asphalt. The stiffness modulus steadily increased as the PE dosage increased. At a doping level of 6%, the stiffness modulus reached 406 MPa and 580 MPa at −18 °C and −24 °C, respectively. While the stiffness modulus of matrix asphalt in the same environment was 382 MPa and 524 MPa, the stiffness modulus of PE-modified asphalt rose by 6.28% and 10.68%, respectively, when compared to matrix asphalt. This is also connected to the net-like cross-linked structure that PE forms in asphalt. The presence of the reticulated cross-linked structure reduced the stress relaxation capacity of the asphalt, resulting in a lower relaxation rate of the PE-modified asphalt compared to the base asphalt for the same temperature as well as for other loads. At the same time, the binding of the mesh cross-linked structure reduced the asphalt’s low-temperature flexibility, making PE-modified asphalt more prone to brittle fracture under temperature stress and other loads, which is not conducive to asphalt’s low-temperature crack resistance. However, because high-grade asphalt has outstanding low-temperature qualities, it may compensate for the shortcomings of waste PE-modified asphalt used in low-temperature situations.

The highest modulus of rigidity specified for asphalt in the Surperpave standard is 300 MPa. The stiffness modulus of the waste PE-modified asphalt surpassed 300 MPa at both −18 °C and −24 °C, as shown in Figure 9. This also demonstrated that the low-temperature fracture resistance of PE-modified asphalt is poor and does not fulfill Surperpave specifications.

The stiffness modulus of SBS-modified asphalt was 204 MPa and 361 MPa at −18 °C and −24 °C, respectively, when the stiffness modulus of PE-modified asphalt was compared. The 6% PE-modified asphalt increased by 99.01% and 60.66% compared to SBS-modified asphalt at −18 °C and −24 °C, respectively. This is also related to the modification mechanism of the two in asphalt; because SBS molecules in asphalt do not form a mesh cross-linked structure, it promotes stress relaxation in the modified asphalt while also improving low-temperature flexibility.

In summary, the low-temperature performance of various asphalt kinds is graded as follows: SBS-modified asphalt is superior to matrix asphalt and PE-modified asphalt.

### 3.4. Analysis of Test Results of Fluorescence Microscopy

**Figure 11:**
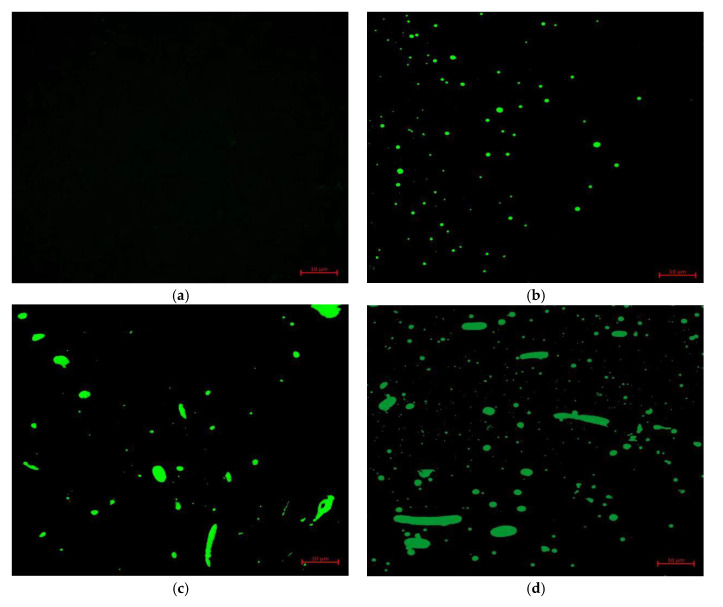

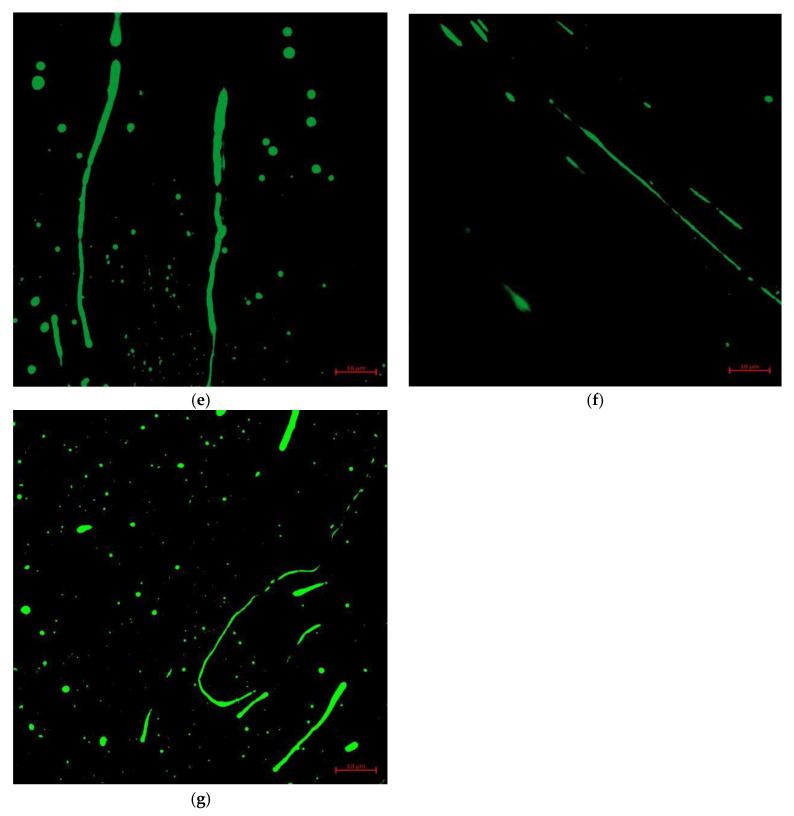
The findings of fluorescence microscopy tests on PE-modified asphalt with varying amounts of doping. (**a**) Base asphalt; (**b**) 1% PE-modified asphalt; (**c**) 2% PE-modified asphalt; (**d**) 3% PE-modified asphalt; (**e**) 4% PE-modified asphalt; (**f**) 5% PE-modified asphalt; (**g**) 6% PE-modified asphalt.

Fluorescence microscopy tests can obtain the distribution of the modifier in the asphalt. The modifier dispersion inside waste PE-modified asphalt with varied dosage doses was examined in this work, and the findings are presented in Figure 11.

Figure 11 shows that when 1% waste PE was mixed in, the waste PE was dispersed in the asphalt in a dotted condition, and at 2%, the dotted waste PE began to polymerize in the asphalt to form a chain. The number of chains as well as their length increased gradually with the increase in doping. This also confirms that adding PE to asphalt can increase its resilience to high-temperature deformation. This is because the chain structure generated by the waste PE in the asphalt can bind the movement of the asphalt, while the PE can absorb the lighter components in the asphalt, reducing their flow capacity in the high-temperature condition. However, the presence of a chain structure will lead to the transformation of plastic deformation into brittle deformation at low temperatures, which will reduce the low-temperature crack resistance of the asphalt.

## 4. Conclusions

The high- and low-temperature performance of the modified asphalt was investigated in this research by incorporating varying volumes of waste PE into high-grade asphalt and performing DSR and BBR tests. The alteration mechanism was also investigated using infrared spectroscopy and fluorescence microscopy, yielding the following results:

(1) The inclusion of waste PE absorbed the asphalt’s lighter components and bonded the asphalt’s flow at high temperatures. When the doping level was 5%, the highest increase in high-temperature performance was obtained.

(2) The waste PE eventually produced a mesh structure in the 90# matrix asphalt as the dosing increased. The existence of this mesh structure is harmful to the creep of asphalt in low-temperature settings, reducing the asphalt’s performance. However, in terms of the balance of the high- and low-temperature characteristics of asphalt, the 1% mix of waste PE causes the modified asphalt to satisfy the low-temperature performance while also improving the high-temperature performance to some level.

(3) Infrared spectroscopy studies revealed that the ability of waste PE to absorb light components in asphalt was proportional to the amount of admixture and the distribution state of waste PE in the asphalt.

(4) Fluorescence microscopy studies revealed that when the waste PE admixture was larger than 2%, chain-like structures progressively formed in the high-grade asphalt. This is caused by the cross-linking of waste PE molecules in the bitumen. This also explains why waste polyethylene has an influence on the high- and low-temperature properties of high-grade asphalt.

## Figures and Tables

**Table 1:** Technical parameters of 90# base asphalt.

Test Items	Unit	Technical Requirements	Test Results	Method
Penetration (25 °C, 5 s, 100 g)	0.1 mm	50~80	72.4	T0604
Softening point (T&B)	℃	35	40.8	T0606
10 °C ductility	Cm	30	84	T0605
15 °C ductility	Cm	100	142	T0605
60 °C power viscosity	Pa·s	140	147	T0620

**Table 2:** Basic indicators of LDPE.

Melting Point °C	Density (g/cm^3^)	Size
112	0.925	0.4~0.5 mm

## References

[1] Kumagai S., Yoshioka T. (2016). Feedstock Recycling via Waste Plastic Pyrolysis. J. Jpn. Pet. Inst..

[2] Masuda T., Kushino T., Matsuda T., Mukai S.R., Hashimoto K., Yoshida S.-I. (2001). Chemical recycling of mixture of waste plastics using a new reactor system with stirred heat medium particles in steam atmosphere. Chem. Eng. J..

[3] Miroslava Š. (2008). Recycling of Waste Industrial Plastics and Possibilities of Its Utilization.

[4] Passamonti F.J., Sedran U. (2012). Recycling of waste plastics into fuels. LDPE conversion in FCC. Appl. Catal. B Environ..

[5] Soni V.K., Singh G., Vijayan B.K., Chopra A., Kapur G.S., Ramakumar S.S.V. (2021). Thermochemical Recycling of Waste Plastics by Pyrolysis: A Review. Energy Fuels.

[6] Wei T., Zhang Z., Zhu Z., Zhou X., Wang Y., Wang Y., Zhuang Q. (2019). Recycling of waste plastics and scalable preparation of Si/CNF/C composite as anode material for lithium-ion batteries. Ionics.

[7] Wang C.Q., Wang H., Liu Y.N. (2015). Separation of polyethylene terephthalate from municipal waste plastics by froth flotation for recycling industry. Waste Manag..

[8] Sharma R., Bansal P.P. (2016). Use of different forms of waste plastic in concrete—A review. J. Clean. Prod..

[9] Babafemi A.J., Šavija B., Paul S.C., Anggraini V. (2018). Engineering properties of concrete with waste recycled plastic: A review. Sustainability.

[10] Ahmad F., Jamal A., Mazher K.M., Umer W., Iqbal M. (2022). Performance evaluation of plastic concrete modified with e-waste plastic as a partial replacement of coarse aggregate. Materials.

[11] Babafemi A.J., Sirba N., Paul S.C., Miah J. (2022). Mechanical and Durability Assessment of Recycled Waste Plastic (Resin8 & PET) Eco-Aggregate Concrete. Sustainability.

[12] Contreras-Marín E., Anguita-García M., Alonso-Guzmán E., Jaramillo-Morilla A., Mascort-Albea E., Romero-Hernández R., Soriano-Cuesta C. (2021). Use of granulated rubber tyre waste as lightweight backfill material for retaining walls. Appl. Sci..

[13] Zhang L., Qiu W., Yang X., Fan H., Zhang S., Zhang A. (2022). Dispersivity Identification and Modification with Lime of Soil in Huaaopao’s Water Conservancy Project. Geotech. Geol. Eng..

[14] Ismail Z.Z., Al-Hashmi E.A. (2008). Use of waste plastic in concrete mixture as aggregate replacement. Waste Manag..

[15] Al-Hadithi A.I., Hilal N.N. (2016). The possibility of enhancing some properties of self-compacting concrete by adding waste plastic fibers. J. Build. Eng..

[16] Khan M.S., Hossain S., Lozano N., Kibria G. (2015). Temporary Lateral Support of a Concrete Retaining Wall Footing using Recycled Plastic Pin. Geo-Congress 2014: Geo-Characterization and Modeling for Sustainability.

[17] Galvão J.C.A., Portella K.F., Joukoski A., Mendes R., Ferreira E.S. (2011). Use of waste polymers in concrete for repair of dam hydraulic surfaces. Constr. Build. Mater..

[18] Haider S., Hafeez I., Ullah R. (2020). Sustainable use of waste plastic modifiers to strengthen the adhesion properties of asphalt mixtures. Constr. Build. Mater..

[19] White G. Evaluating recycled waste plastic modification and extension of bituminous binder for asphalt. Proceedings of the Eighteenth Annual International Conference on Pavement Engineering, Asphalt Technology and Infrastructure.

[20] Fang C., Zhang Y., Yu Q., Zhou X., Guo D., Yu R., Zhang M. (2013). Preparation, Characterization and Hot Storage Stability of Asphalt Modified by Waste Polyethylene Packaging. J. Mater. Sci. Technol..

[21] Boom Y.J., Xuan D.L., Enfrin M., Swaney M., Masood H., Pramanik B.K., Robert D., Giustozzi F. (2023). Engineering properties, microplastics and emissions assessment of recycled plastic modified asphalt mixtures. Sci. Total Environ..

[22] Ren S., Liu X., Jing R., Gao Y., Lin P., Erkens S. (2023). Investigating the rheological properties and compatibility behaviours of RET/PE and WR/CR/SBS compound-modified bitumen. Road Mater. Pavement Des..

[23] Ma Y., Wang S., Zhang M., Jiang X., Polaczyk P., Huang B. (2023). Weather aging effects on modified asphalt with rubber-polyethylene composites. Sci. Total Environ..

[24] Ren S., Liu X., Lin P., Jing R., Erkens S. (2022). Toward the long-term aging influence and novel reaction kinetics models of bitumen. Int. J. Pavement Eng..

[25] Xiao R., Shen Z., Polaczyk P., Huang B. (2023). Thermodynamic Properties of Aggregate Coated by Different Types of Waste Plastic: Adhesion and Moisture Resistance of Asphalt-Aggregate Systems. J. Mater. Civ. Eng..

[26] Behnood A., Gharehveran M.M. (2019). Morphology, rheology, and physical properties of polymer-modified asphalt binders. Eur. Polym. J..

[27] Pasetto M., Baliello A., Pasquini E., Poulikakos L. (2022). Dry addition of recycled waste polyethylene in asphalt mixtures: A laboratory study. Materials.

[28] Dalhat M.A., Al-Abdul Wahhab H.I. (2017). Performance of recycled plastic waste modified asphalt binder in Saudi Arabia. Int. J. Pavement Eng..

[29] Yu Y., Shi C., Xu G., Yao Z., Wang T., Wu Y., Yi X., Wang H., Yang J. (2022). Application of nanoindentation in asphalt material aging and characterization of actual pavement aging. Constr. Build. Mater..

[30] Cheng Y., Wang H., Wang W., Liang J. (2023). Rheological evolution mechanisms of asphalt binder and mastic under freeze-thaw cycles. Constr. Build. Mater..

[31] Dittrich M., Sibler S. (2005). Cell surface groups of two picocyanobacteria strains studied by zeta potential investigations, potentiometric titration, and infrared spectroscopy. J. Colloid Interface Sci..

[32] Wu G., Zhang H., Sun J., Yu T. (2021). Comparative analysis on rheological characteristics of different modified asphalt based on DSR and BBR evaluation. J. Eng. Des. Technol..

[33] Wen Y., Guo N., Wang L., Jiao B., Li W. (2021). Rheological properties and microscopic mechanism of rock asphalt composite modified asphalts. Constr. Build. Mater..

